# Termite mounds contain soil-derived methanotroph communities kinetically adapted to elevated methane concentrations

**DOI:** 10.1038/s41396-020-0722-3

**Published:** 2020-07-24

**Authors:** Eleonora Chiri, Chris Greening, Rachael Lappan, David W. Waite, Thanavit Jirapanjawat, Xiyang Dong, Stefan K. Arndt, Philipp A. Nauer

**Affiliations:** 1 Department of Microbiology, Biomedicine Discovery Institute, Monash University, Clayton, VIC 3800 Australia; 2 School of Ecosystem and Forest Sciences, University of Melbourne, Richmond, VIC 3121 Australia; 3 School of Biological Sciences, Monash University, Clayton, VIC 3800 Australia; 4 School of Biological Sciences, University of Auckland, Auckland, New Zealand; 5 School of Marine Sciences, Sun Yat-Sen University, Zhuhai, 519082 China; 6 School of Chemistry, Monash University, Clayton, VIC 3800 Australia

**Keywords:** Soil microbiology, Biogeochemistry

## Abstract

Termite mounds have recently been confirmed to mitigate approximately half of termite methane (CH_4_) emissions, but the aerobic CH_4_ oxidising bacteria (methanotrophs) responsible for this consumption have not been resolved. Here, we describe the abundance, composition and CH_4_ oxidation kinetics of the methanotroph communities in the mounds of three distinct termite species sampled from Northern Australia. Results from three independent methods employed show that methanotrophs are rare members of microbial communities in termite mounds, with a comparable abundance but distinct composition to those of adjoining soil samples. Across all mounds, the most abundant and prevalent methane monooxygenase sequences were affiliated with upland soil cluster α (USCα), with sequences homologous to *Methylocystis* and tropical upland soil cluster (TUSC) also detected. The reconstruction of a metagenome-assembled genome of a mound USCα representative highlighted the metabolic capabilities of this group of methanotrophs. The apparent Michaelis–Menten kinetics of CH_4_ oxidation in mounds were estimated from in situ reaction rates. Methane affinities of the communities were in the low micromolar range, which is one to two orders of magnitude higher than those of upland soils, but significantly lower than those measured in soils with a large CH_4_ source such as landfill cover soils. The rate constant of CH_4_ oxidation, as well as the porosity of the mound material, were significantly positively correlated with the abundance of methanotroph communities of termite mounds. We conclude that termite-derived CH_4_ emissions have selected for distinct methanotroph communities that are kinetically adapted to elevated CH_4_ concentrations. However, factors other than substrate concentration appear to limit methanotroph abundance and hence these bacteria only partially mitigate termite-derived CH_4_ emissions. Our results also highlight the predominant role of USCα in an environment with elevated CH_4_ concentrations and suggest a higher functional diversity within this group than previously recognised.

## Introduction

Termites are mound-building eusocial insects that live in colonies throughout the tropics and subtropics. These organisms completely degrade lignocellulose in a process primarily mediated by anaerobic symbiotic microorganisms in their hindgut [1]. During this process, hydrogenotrophic methanogens produce substantial amounts of methane (CH_4_) that is emitted from the termite into the atmosphere [2–4]. Production rates vary by three to four orders of magnitude depending on the termite species and their dietary preferences (i.e. wood-, grass-, soil- or fungus-feeding) [1, 4, 5]. Current models suggest that termites are responsible for between 1 and 3% of global CH_4_ emissions to the atmosphere [6].

Aerobic CH_4_ oxidizing bacteria (methanotrophs) significantly mitigate emissions of CH_4_ from termites [7]. Methanotrophs gain carbon and energy by oxidising CH_4_ to carbon dioxide, with the first step in this reaction being catalysed by particulate and soluble methane monooxygenases [8]. It is controversial whether termite hindguts harbour such organisms; while *Methylocystis* spp. were recently isolated from termites [9], other studies could not detect methanotroph functional gene markers or measurable amounts of ^14^CO_2_ during ^14^CH_4_ incubation experiments [10]. We also observed that the addition of inhibitors of CH_4_ oxidation did not increase direct termite CH_4_ emissions [7]. However, many termite colonies construct large mounds built from soil material or build their nest in soil, which is generally a sink for atmospheric CH_4_ [11]. While results from incubation experiments of mound material were conflicting [12–14], we recently presented clear evidence of widespread CH_4_ oxidation in North Australian termite mounds [7]: results from three different in situ methods to measure CH_4_ oxidation in mounds confirmed that methanotrophs mitigate between 20 and 80% of termite-derived CH_4_ before emission to the atmosphere. However, the community composition and CH_4_ oxidation kinetics of the methanotrophs responsible remain largely unknown.

Compared with soils, methanotrophs inhabiting termite mounds have received little attention. Ho et al. [14] investigated mound material of the African fungus-feeding termite *Macrotermes falciger* using a *pmoA*-based diagnostic microarray approach. Community composition differed between mound and soil, and at some locations within the large compartmentalised mounds. Slurry incubations confirmed potential CH_4_ oxidation at high and low CH_4_ concentrations. The mound community was reportedly dominated by gammaproteobacterial methanotrophs of the Jasper Ridge 3 (JR3) cluster, while the functional gene of the soluble methane monooxygenase could not be detected and nor could Verrucomicrobia and Methylomirabilota (NC10) methanotrophs. Beside this pioneering work, the mound methanotroph communities of no other termite species has been investigated. However, large differences might exist between mounds of different termite species given factors such as their different dietary preferences, as well as the impressive variety of mound sizes, shapes and internal structures [15, 16]. Reflecting this, in situ studies have shown that there is a large variation in methanotroph activity in mounds both within and between species; for example, *Tumulitermes pastinator* mounds appear to be largely inactive and still a high fraction of termite-derived CH_4_ can be oxidised in soil beneath mounds, due to facilitation of CH_4_ transport within the mound [7]. It remains unclear whether differences in methanotroph community abundance or composition account for these activity differences.

In this work, we aimed to resolve these discrepancies by conducting a comprehensive analysis of the composition and kinetics of the methanotroph communities within termite mounds of Australian termite species. Three mound-building termite species were selected, the wood-feeding *Microcerotermes nervosus* (Mn), soil-interface-feeding *Macrognathotermes sunteri* (Ms) and grass-feeding *Tumulitermes pastinator* (Tp), which represent the three main feeding groups present in Australia [17]. Mounds of these species were previously confirmed to oxidise a high fraction of termite-produced CH_4_ [7]. We comprehensively assessed the abundance, diversity and composition of methanotroph communities within 17 mounds and a subset of adjoining soils; to do so, we combined metagenomic sequencing with amplicon sequencing and quantitative PCR (qPCR) of both the 16S rRNA gene and the *pmoA* marker gene, encoding a subunit of the particulate methane monooxygenase present in most methanotrophs [18]. In parallel, we performed in situ studies using gas push–pull tests (GPPTs) to derive the apparent kinetic parameters of CH_4_ oxidation. We demonstrate that methanotrophic communities in termite mounds are derived from surrounding soils, but are compositionally and kinetically distinct, and primarily comprise upland soil cluster α (USCα) alphaproteobacteria with an apparent medium affinity for CH_4_.

## Materials and methods

### Field sites and sampling

Field tests and sampling were performed in April and May 2016 in a coastal savanna woodland on the campus of Charles Darwin University in Darwin, Northern Territory, Australia (12.370° S, 130.867° E). The site is described in detail in Nauer et al. [19], and is representative of large areas of tropical northern Australia in terms of vegetation and soil conditions. For this study, 29 termite mounds were first subject to in situ CH_4_ oxidation measurements using GPPTs (described below). For further investigations following field measurements, we selected 17 mounds of an appropriate size for processing in the laboratory (initially 18, but one was damaged during transport and had to be discarded). These mounds were first excavated but kept intact to measure internal structure, volume, densities and porosities as previously described [19]. They were then deconstructed to (1) sample termites for species identification, (2) collect mound material for physicochemical analyses and gravimetric water content measurements and (3) collect mound material for molecular analyses of methanotrophic community. For termite species identification, soldiers were individually picked and stored in pure ethanol as previously described [19]. For gravimetric water content measurements, ~200 g of mound material from both core and periphery locations were subsampled and oven-dried at 105 °C for >72 h; subsamples were measured before and after drying and the water content calculated based on mass loss. Subsamples for physicochemical parameters were oven-dried at 60 °C for 72 h, carefully homogenised into a composite sample for each termite species and location, and sent to an external laboratory for analyses according to standard protocols (CSBP Laboratory, Bibra Lake WA, Australia). For community analysis, mound and soil material was collected under sterile conditions using bleach- and heat-sterilised spatulas, and immediately stored in autoclaved 2 ml centrifuge tubes at −20 °C. For each of the 17 mounds, we collected pooled samples of mound core and periphery material derived from three different spots. Mound cores were sampled from within 20–30 cm from the approximate centroid of mound, whereas mound periphery was collected from the outer 5–10 cm of the mound. For a subset of the investigated mounds, soil was collected from beneath the mound immediately after mound excavation, and from the surrounding soil within a 1–2 m radius from the mound. This resulted in 34 mound and 14 soil samples processed for molecular analyses.

### Genomic DNA extraction

Each individual sample of mound material and soil was homogenised. DNA was extracted from 0.25 to 0.5 g of each sample using the PowerLyzer PowerSoil DNA Isolation Kit (Qiagen, US), according to the manufacturer instructions. The purity and integrity of the DNA extracts was verified by spectrophotometry (NanoDrop ND-1000 spectrophotometer, Nanodrop Technologies Inc., USA) and PCR amplification of 16S rRNA genes. Good yields of high-quality, amplifiable genomic DNA were obtained from all 48 samples. PCR-grade water was extracted as a negative control.

### Quantitative PCR

qPCR assays were used to estimate the abundance of the total bacterial community and methanotroph community. Methanotroph abundance was estimated by amplifying the *pmoA* gene using previously described degenerate primers (A189f 5′-GGNGACTGGGACTTCTGG-3′ and mb661 5′-CCGGMGCAACGTCYTTACC-3′) [20, 21] and cycling conditions [22]. These primers were chosen as a compromise to cover methanotrophs from environments with elevated CH_4_ concentrations and those of upland soil communities, while minimising amplification of closely related ammonium-oxidizing bacteria likely to be of high abundance in termite mounds [23]. Total bacterial abundance was estimated by amplifying the 16S rRNA gene using degenerate primers (515FB 5′-GTGYCAGCMGCCGCGGTAA-3′ and 806RB 5′-GGACTACNVGGGTWTCTAAT-3′) and cycling conditions as previously described [24–26]. Gene copy numbers were determined using a LightCycler 480 real-time PCR system (Roche, Basel, CH). Individual reactions contained 1 × PowerUp SYBR Green Master Mix (Thermo Fisher Scientific), 400 µM of each primer and 1 µl of diluted environmental DNA mixed to a final volume of 20 µl. Thermal profiles were adapted from those used for previous PCRs and included an acquisition step of 85 °C for 30 s at the end of each amplification cycle. Melting curve analysis was performed as follows: 95 °C for 15 s, 60 °C for 60 s, 95 °C for 30 s and 60 °C for 15 s. For each assay (96-well plate), duplicate serial dilutions of quantified DNA extract from *Methylosinus trichosporium* strain OB3b [27] were used for calibration curves to quantify 16S rRNA genes or *pmoA* genes. Each sample was analysed in triplicate, and a total of three assays were required for each gene to include all the samples. Amplification efficiencies calculated from the slopes of calibration curves were >70% and *R*^2^ values were >0.98. Using the copy numbers obtained, the total methanotroph community was estimated by assuming an average of two *pmoA* copies per cell. It is common for methanotrophs to possess two of the *pmoA* gene, and this average has been previously applied in studies investigating abundance of methanotrophs in upland soils [28, 29]. The total bacterial community was estimated by assuming an average of 4.2 16S rRNA gene copies per cell [30]. No significant amplification of the negative water extractions was observed in either the *pmoA* or 16S rRNA gene qPCR assays.

### PmoA amplicon sequencing

The structure of the methanotroph community within each sample was primarily inferred from amplicon sequencing of community *pmoA* genes. Amplification reaction mixtures (25–50 µl final volume) were prepared using 1 µl of DNA extract as template, 1 × PCR buffer, 0.2 mM of each primer, 0.25 mM deoxynucleoside triphosphates and 0.025 U µl^−1^ of Taq polymerase (Takara Biotechnology Ltd., Japan). Different dilutions (from undiluted to 1:100 dilution in PCR-grade water) of DNA extracts were used as template during amplification, and the dilution resulting in the highest yield and quality of PCR product was used for further analyses. DNA extracts of all samples were sent to the Australian Genome Research Facility (Brisbane, QLD) for preparation of *pmoA* gene amplicon libraries using the above primer sets (A189f and mb661) and thermal conditions. Subsequent amplicon sequencing was performed on a MiSeq DNA-sequencing platform using a 600-cycle MiSeq Reagent Kit v3 (Illumina, San Diego, CA). Sequencing yielded 5,594,739 paired-end sequences, of which 3,078,335 passed quality checks and data processing, and were used for subsequent analyses. Sequence read counts spanned three orders of magnitude (10^5^–10^2^), with 54% of the samples exhibiting read counts above the average read count value (>65 K) and most mound periphery samples having read counts below 10 K. Six samples with read counts below 1000 were excluded from subsequent analyses. Sequencing data were processed according to our previously published pipeline [31], with minor modifications. Briefly, reads were 3′-trimmed to remove ambiguous or low-quality endings, then merged and primer-site trimmed. Quality filters included an amplicon-size selection (471 nt) and the removal of amplicons containing stop codons (i.e. TAA, TAG and TGA). Sequences were also checked for correct open reading frames using the FrameBot tool (http://fungene.cme.msu.edu/FunGenePipeline/framebot/form.spr). The centroid clustering method [32] identified 25 operational taxonomic units (OTUs) that shared 86% nucleotide sequence similarity [33] with sequences from a curated *pmoA* gene database derived from Dumont et al. [34]. Phylogenetic distances of the assigned OTUs in relation to reference *pmoA* sequences were assessed as previously described [31]. The phylogenetic tree of amino acid-deduced *pmoA* sequences was constructed in Seaview4 [35] using the maximum-likelihood method and the LG empirical amino acid substitution model, which showed the lowest Akaike information criterion (AIC) during substitution model testing, and was bootstrapped using 100 bootstrap replicates. All sequences affiliated with methanotrophs, with no clear *amoA* nor *pxmA* sequences detected.

### 16S rRNA gene amplicon sequencing

For each sample, the V4 hypervariable region of the 16S rRNA gene was amplified using the universal Earth Microbiome Project primer pairs F515 and R806 [24]. Samples were subject to Illumina MiSeq paired-end sequencing at the Australian Centre for Ecogenomics, University of Queensland. Using the QIIME 2 platform [36], the resultant raw sequences were subject to quality filtering, primer trimming, denoising and singleton removal. The final dataset contained 9998 high-quality 16S rRNA gene amplicon sequence variants (ASVs). For alpha diversity analysis, observed richness, Shannon index and Inverse Simpson index were analysed using the package phyloseq v1.30 [37]. For beta diversity analysis, Bray–Curtis community dissimilarity was visualised using non-parametric multidimensional scaling ordinations (nMDS) in phyloseq v1.30 [37]. To estimate the proportion of methanotrophs in the community via the 16S rRNA gene amplicon dataset, the obtained ASVs were aligned to a reference database containing 16S rRNA gene sequences from methanotrophs and related non-methanotrophic isolates and MAGs (Table S1). The V4 hypervariable region of the 16S rRNA gene was extracted from the reference sequences using QIIME 2 (extract-reads function in the feature-classifier plugin). Identical reference sequences were collapsed before alignment of V4-derived ASVs to this database with the global aligner VSEARCH v2.13.6 [38] (–id 0.90, –maxaccepts 5, –top_hits_only). The evolutionary relationships of the reference sequences and the 380 ASVs with a percentage sequence identity ≥90% were then visualised in phylogenetic trees to assess whether they most closely branch with known methanotrophs. It was found that 27 ASVs branched together with methanotrophic bacteria, all from the *Methylocystaceae* and *Hyphomicrobiaceae* families, whereas the remaining sequences were assumed to be from non-methanotrophic taxa (Table S2). A phylogenetic tree of these ASVs was constructed in Seaview4 [35] using a GTR-based model and bootstrapped with 100 replicates. The relative abundance of these ASVs was calculated compared with the rest of the 16S rRNA gene dataset. Note that this analysis cannot detect divergent uncultured methanotrophs with unknown 16S rRNA gene sequences, most notably TUSC.

### Phylogenetic and diversity analyses

Alpha and beta diversity calculations, as well as read count normalisation of the *pmoA* sequences, were performed with the package phyloseq v1.12.2 [37] from the open source software Bioconductor. To account for differences in numbers of reads between samples, we rarefied OTU counts to an even sampling depth of 1611 read counts. Chao1, Shannon and Inverse Simpson indices were computed to assess the alpha diversity of MOB communities. Beta diversity of methanotroph communities was measured using the phylogenetic metric Unifrac weighted by the relative abundance of individual OTUs [39]. Distances between sample groups were visualised using nMDS ordinations. To determine whether the observed between-group distances were statistically significant, we performed permutational multivariate analysis of variance (PERMANOVA) with the software PRIMER-E v7 (PRIMER-E Ltd., Plymouth, United Kingdom). Negative binomial models were performed on the non-rarefied OTU dataset to assess the differential abundance of bacterial OTUs between sample groups, and the false discovery rate approach was used to account for multiple testing.

### Shotgun metagenome sequencing and analysis

DNA extracts of mound cores and adjacent soils were pooled for each of the three termite species investigated (six samples in total). These samples were subject to metagenomic shotgun library preparation using the Nextera XT DNA Sample Preparation Kit (Illumina Inc., San Diego, CA, USA) at the Australian Centre for Ecogenomics. Sequencing was performed on an Illumina NextSeq500 platform with a 2 × 150 base pair High Output run. Raw metagenomic paired-end reads were inspected for quality with FastQC v0.11.7 [40] and MultiQC v1.0 [41]. BBDuk v38.51 from the BBTools suite [42] was used to trim adaptor sequences, remove reads aligning to the PhiX genome and trim low-quality bases (minimum quality score 20) and discard reads <50 bp in length. After preprocessing, an average of 91.2% of the raw read pairs per sample remained (76.6 M paired-end reads). To calculate the abundance of *pmoA* and *mmoX* (encoding for a subunit of the soluble methane monooxygenase) genes in the short read data, the pre-processed reads were aligned with the blastx function of DIAMOND v0.9.24 [43] against a manually curated database of deduced amino acid sequences for each gene (PmoA and MmoX). The employed PmoA database is the same one we used for the study of the *pmoA* gene amplicons and comprises sequences from methanotroph isolates, draft genomes and uncultivated sequence clusters. Forward reads at least 140 bp length were aligned with a query coverage threshold of 80% and a percentage identity threshold of 60% to PmoA and MmoX. Hits to the PxmA sequences were not included in subsequent estimates of methanotroph abundance. The abundance of each gene was converted to reads per kilobase million (RPKM). These adjusted gene abundance values were used to estimate the proportion of the community carrying each gene by normalising to the abundance for universal single-copy genes. The same reads were aligned to each of the 14 single-copy ribosomal marker genes available in SingleM (https://github.com/wwood/singlem) with DIAMOND (query coverage 80%). Alignments with a bitscore below 40 were removed, and the counts converted to RPKM and averaged across the 14 genes. The RPKM values for *pmoA* and *mmoX* were divided by the average RPKM value of the single-copy genes for each sample, and it was assumed that each methanotroph genome encoded two copies of *pmoA* per genome on average.

### Genome assembly and analysis

Quality-filtered paired-end and singleton reads were assembled from each sample using metaSPAdes v3.13.0 [44] with k-mer sizes 43, 55, 77, 99 and 121. Contigs shorter than 1500 bp were removed from the assembly using seqmagick v0.6.1 (https://fhcrc.github.io/seqmagick/), then paired-end reads were mapped to the remaining contigs using BBMap to obtain coverage profiles. Differential coverage binning was performed for each sample using MetaBAT v2.12.1 [45], MaxBin v2.2.4 [46] and CONCOCT v1.1 [47]. All tools were run using default parameters, with the exception of MetaBAT2 where the minimum contig size was set to 1500 bp. Within each sample, bins were dereplicated using DAS_Tool v1.0 [48], resulting in a total of 136 bins. The reduced set of bins were then refined using a modified version of the VizBin protocol [49], performing a t-SNE projection which included coverage as a feature and weighted it to account for 50% of the distance calculation. Projections were performed for each sample and contigs with uncertain placement were manually removed from bins. The refined set of bins were combined into a single collection, assessed for completeness and contamination using CheckM v1.0.13 [50], then dereplicated using dRep v2.3.2 [51]. A total of 55 bins, with at least 50% completeness, were retained after this process. Taxonomy was assigned to all bins using the GTDB-TK v0.2.2 software [52]. Inspection of the classification summary revealed a single bin (MAG-47) classified as a member of the proteobacterial genus *Methylocapsa* (*Alphaproteobacteria*; *Rhizobiales*; *Beijerinckiaceae*), which was explored in detail. A robust phylogeny was inferred for this bin by extracting the GTDB-TK alignment for 135 bacterial genomes from the family *Beijerinckiaceae*. This selection was augmented with the MAG USCα AHI (VDMG00000000.1) [53] and confirmed to include the species *M. acidiphila* B2 (GCF_000427445.1), which was used in the original characterisation of the USCα *pmo* operon [54]. A selection of 20 bacterial species was randomly sampled from families within the order *Rhizobiales*, excluding *Beijerinckiaceae*, for use as an outgroup. Phylogenetic inference was performed using IQ-Tree v1.6.12 [55] with the Le and Gascuel model of amino acid evolution [56] with empirically determined amino acid frequencies and Gamma-distributed rate heterogeneity (LG + F + G). Bootstrap support was calculated using 100 resamplings, and the tree visualised using iToL v4 [57]. For metabolic pathway reconstruction of MAG-47, the genome was firstly annotated using MetaErg v1.2.2 [58]. The predicted amino sequences were then used as query for identification of key metabolism markers via METABOLIC v2.0 [59].

### Gas push–pull tests

The GPPT was used to estimate in situ activity coefficients as described previously [7, 60]. Michaelis–Menten parameters estimated from in situ methods are integrated measures across a large mass of substrate and are thus better suited to characterise the kinetic potential of whole microbial communities in heterogeneous systems than laboratory microcosms, which suffer from inevitable sampling bias [61]. In brief, a gas mixture containing laboratory air, ~900 µl l^−1^ of CH_4_, and ~0.1 l l^−1^ argon (Ar) was injected at a rate of ~0.5 l min^−1^ into the lower centre of the termite mounds and then immediately extracted from the same location at the same flow rate. During extraction, the injected gas mixture was gradually diluted with termite mound air down to background levels; the tracer Ar accounted for this dilution due to its similar transport behaviour to CH_4_. A timeseries of CH_4_ and Ar concentrations was collected during the 24 min injection phase, and the 36 min extraction phase. Concentrations of CH_4_ were measured quasi-continuously (frequency of 1 Hz) using a field-portable spectrometer (Fast Greenhouse Gas Analyser Los Gatos Research, Mountain View, CA). For Ar, discrete samples were collected at fixed intervals during injection (*n* = 3) and extraction (*n* = 10–12), as well as prior to injection to determine background levels. Argon concentrations were analysed on a customised gas chromatography system (SRI 8610, SRI Instruments, Torrance, CA) with an external thermal conductivity detector (VICI Valco Instruments Co., Houston, TX). To improve separation of Ar, oxygen was removed from the sample gas stream prior to separation with a manually packed Pd-Al catalyst column [7, 62].

### Kinetic analysis

First-order rate coefficients of CH_4_ oxidation (activity coefficient *k*) were estimated from GPPTs via the slope of the logarithm of relative CH_4_ vs Ar concentrations, plotted against a transformed reaction time, according to the plug-flow reactor model for simplified GPPT analysis [63]. These data also allowed the calculation of reaction rates at different CH_4_ concentrations from segments of the extraction timeseries, and thus the estimation of apparent Michaelis–Menten parameters [64]. The running average of CH_4_ concentrations (*C*_CH4_) over three consecutive extraction samples was multiplied with the corresponding activity *k* from the logarithmic plot to calculate an individual reaction rate (*R*_ox_) for each segment. The apparent Michaelis–Menten parameters (*K*_m_ and *V*_max_) were then estimated for each individual GPPT, and for all combined pairs of concentrations and reaction rates, by fitting the Michaelis–Menten model *R*_ox_ = *V*_max_ × *C*_CH4_/(*K*_m_ + *C*_CH4_) to the data using the non-linear regression routine nls() in R [65]. The AIC was calculated and compared with a linear regression model of *R*_ox_ vs *C*_CH4_; if the AIC of the linear model was lower, no Michaelis–Menten parameters were reported. Cell-specific reaction rates were calculated from overall GPPT reaction rates based on total mound dry mass [7], divided by *pmoA* copy numbers and assuming two gene copies of *pmoA* per cell. Correlations between kinetic parameters (*k*, *K*_m_, *V*_max_), gene abundance (*pmoA* and 16S rRNA genes), and physical mound parameters (mound micro- and macro-porosity, total volume and water content) were tested for significance using linear regression (Table S3), after transformations (sqrt for kinetic parameters, log for gene abundances) and removal of outliers as indicated by diagnostic plots (qq- and Cook’s distance). Stepwise parameter elimination was performed using the step() function in R [65].

## Results and discussion

### Methanotrophic bacteria are in low abundance in termite mounds and associated soils

**Fig. 1:**
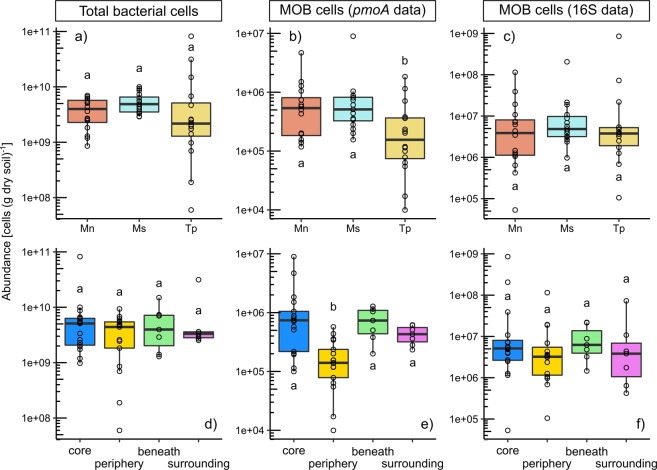
Summary of differences in the abundance of total bacteria and methanotrophs. Samples are grouped according to termite species (**a**–**c**) and sampling location (**d**–**f**). **a**, **d** show total bacterial cells estimated from 16S rRNA gene qPCR, assuming on average 4.2 16S rRNA gene copies per cell. **b**, **e** show methanotroph cells estimated from *pmoA* qPCR, assuming 2 *pmoA* copies per cell. **c**, **f** show methanotroph abundance estimated from 16S rRNA gene qPCR, using the ratio of identified methanotrophs to total 16S rRNA gene copies. Different letters denote significant differences in abundance between sample groups (*p* < 0.05, Kruskal–Wallis and Dunn test). Mn *Microcerotermes nervosus* mounds, Ms *Macrognathotermes sunteri* mounds, Tp *Tumulitermes pastinator* mounds, core termite mound core, periphery termite mound periphery, beneath soil beneath mound, surrounding soil surrounding mound, MOB methane-oxidising bacteria.

qPCR was used to estimate the abundance of the methanotroph community (via *pmoA* gene) and total bacterial community (via 16S rRNA gene) in mound and soil samples. Bacterial abundance was consistently high (av. 6.4 × 10^9^ cells per gram of dry soil; range 6.0 × 10^7^–8.2 × 10^10^) and did not significantly differ between sample locations (Figs. 1a, d and S1); an earlier study found higher microbial biomass in the mound compared with soil [66], but this may reflect different methodologies applied to each substrate. In contrast, methanotroph cell abundance was relatively low across the samples (av. 1.5 × 10^6^ copies per gram of dry sample material; range: 1.0 × 10^4^–9.0 × 10^6^) and just 0.016% that of total bacterial cells (range: 0.0004–0.10%) (Figs. 1b, e and S1). Such values are comparable to those previously reported for the abundance of *pmoA* genes in upland soils that mediate atmospheric CH_4_ oxidation (~10^6^ copy number, ~0.01% relative abundance [29]). However, while *pmoA*-based methods are a standard method for inferring methanotroph abundance and enable comparisons with previous studies [29, 31, 67], they can potentially underestimate the CH_4_-oxidizing community given *pmoA* primer mismatches can result in low amplification of certain methanotroph groups such as USCα [18].

Two other methods were therefore used to predict the abundance of the methanotroph community. We used 16S rRNA gene amplicon sequencing, in conjunction with phylogenetic analysis, to identify CH_4_-oxidising taxa within the context of the total bacterial and archaeal community. Overall, the bacterial community of termite mound samples had lower richness and distinct composition to soil samples, including an increased relative abundance of *Actinobacteria* and decreased relative abundance of *Gammaproteobacteria* across the mounds of all three termite species sampled (Fig. S2; Table S4). On average, 0.24% of the community (range: 0.004–2.37%) affiliated with known methanotrophs, all within the class *Alphaproteobacteria* (Figs. 1c, f and S1; Table S4). In parallel, we performed shotgun metagenome sequencing and used homology-based searches of quality-filtered short reads to estimate the abundance of genes encoding particulate (*pmoA*) and soluble (*mmoX*) methane monooxygenases relative to single-copy ribosomal marker genes; on average, 0.21% of the community (range: 0.13–0.53%) are predicted to encode methane monooxygenases, with 82% of the hits to the particulate type (Table S2). While the results of 16S rRNA gene and metagenome analyses are concordant, the values obtained are an order of magnitude higher than estimates based on *pmoA* qPCR data. It is unclear which results are most reliable given all three methods have intrinsic detection biases that can lead to the underestimation and overestimation of particular methanotroph groups. However, regardless of the method used to infer their relative abundance, methanotrophs appear to be members of the rare biosphere in mounds and soils alike despite the elevated CH_4_ concentrations available in the mound environment.

Some differences in methanotroph abundance were observed between sample locations and termite species (Table S3). Based on *pmoA* qPCR, methanotroph abundance was 3.5-fold higher in mound core and 1.5-fold higher in soil beneath than in surrounding soil, though differences were below the threshold of significance (Fig. 1e). In contrast, methanotroph abundance was significantly lower in mound periphery samples of all species (*p* < 0.01; Kruskal–Wallis and Dunn test; Fig. 1e) and in mound samples of *T. pastinator* compared with the other two species tested (*p* < 0.01; Kruskal–Wallis and Dunn test; Fig. 1b); the latter observation is in line with the finding that CH_4_ oxidation occurs at low rates in *T. pastinator* mound material [7]. Similar but weaker trends were also observed based on estimating methanotroph abundance via 16S rRNA gene amplicon sequencing (Fig. 1c, f). We performed linear regression on how environmental parameters correlated with methanotroph abundance, including stepwise elimination of parameters (Table S3). Micro-porosity and sampling location were the only significant parameters remaining (*p* < 0.001 for location and micro-porosity, *p* = 0.08 for water content). With the exception of macro-porosity in periphery samples, other physical parameters did not correlate significantly with methanotroph or total bacterial abundance (Table S3). This suggests that denser mound material, as found in mound periphery and *T. pastinator* mounds, limits methanotroph abundance and activity [7, 19]. These differences may also reflect the relatively harsh conditions in the mound periphery, with its strong fluctuations of temperature and water content, compared with the core with termite-engineered homoeostasis [68, 69].

### Termite mounds contain compositionally distinct methanotroph communities that are derived from surrounding soils

**Fig. 2:**
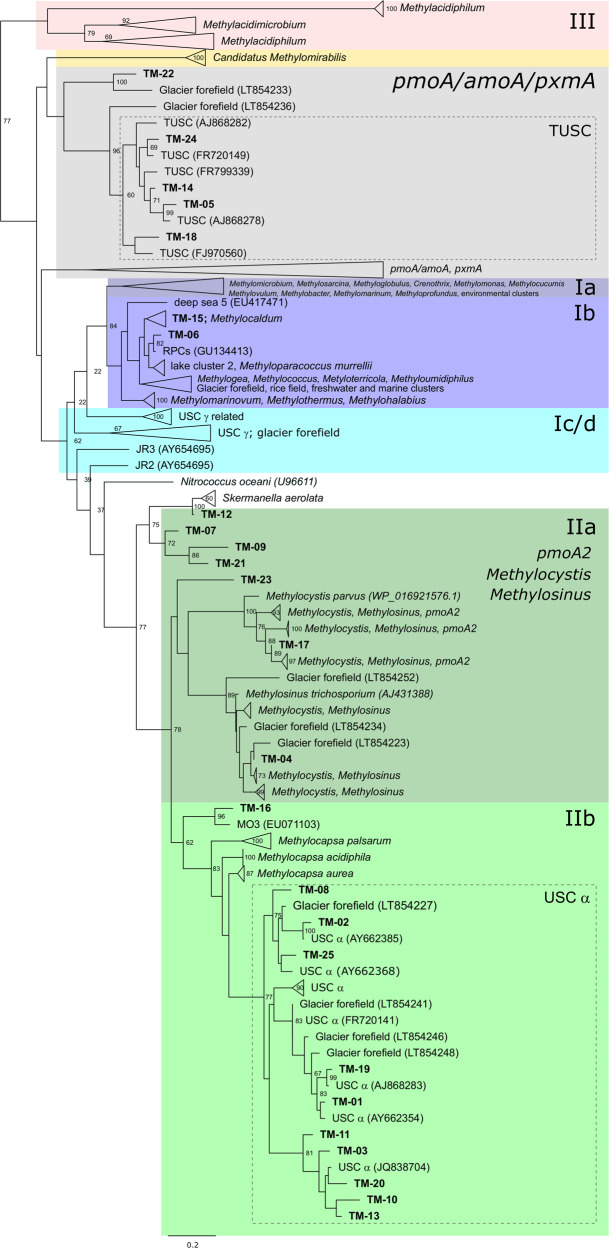
Maximum-likelihood tree showing the phylogenetic affiliation of the deduced amino acid *pmoA* gene sequences of 25 operational taxonomic units (OTUs), in relation to uncultivated methanotrophic clusters and methanotroph isolates. The 25 OTUs are depicted in bold and numbered according to decreasing relative abundance among all samples. The tree was built using the LG empirical amino acid substitution model and bootstrapped using 100 bootstrap replicates. Node numbers indicate bootstrap branch support ≥60. Genbank accession numbers for the sequences at individual node tips are given in parentheses. The scale bar displays 0.2 changes per amino acid position.

**Fig. 3:**
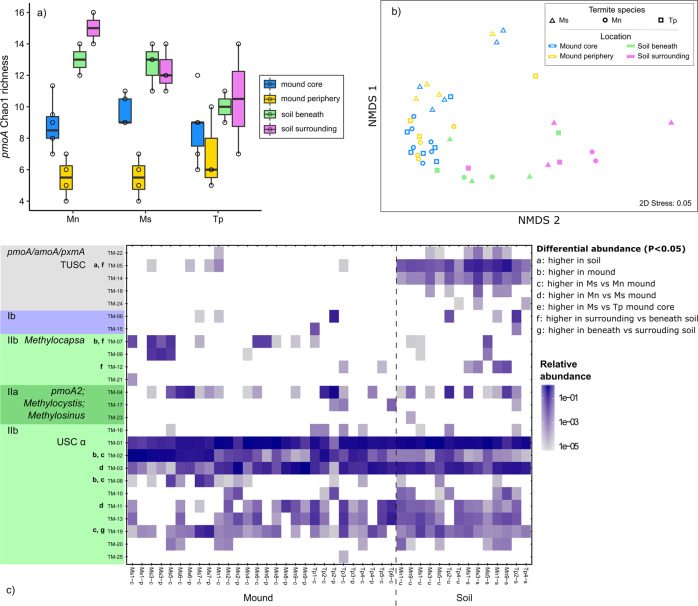
Community composition of CH_4_ oxidising bacteria (methanotrophs) of mounds and adjoining soils. **a** Estimated richness of the methanotroph community, based on Chao1 index of the *pmoA* gene as determined by amplicon sequencing. **b** Non-metric multidimensional scaling (nMDS) ordination of the methanotrophic community structure (beta diversity) measured by weighted UniFrac distance metric of the *pmoA* gene, and differentiation according to termite species and sample location. **c** Heatmap of the relative abundance of the *pmoA* OTUs in all samples. OTUs are ordered according to their position on the phylogenetic tree shown in Fig. 2. Differential abundance of *pmoA* OTUs between sample groups was assessed from negative binomial models of the OTU read counts; *p* values were corrected with the false discovery rate approach to account for multiple testing. Only significant tests are shown (*p* < 0.05).

We analysed the diversity and composition of the methanotroph community. To do so, we focused on *pmoA* amplicon sequencing given this method enables higher-resolution methanotrophic community analysis, but verified findings using 16S rRNA gene amplicon and metagenomic sequencing. Across the samples, 25 OTUs were detected (Fig. 2). Observed and estimated richness of these OTUs was higher in soil samples compared with mound samples (*p* < 0.001; Wilcoxon rank-sum test), and mound core compared with periphery (*p* < 0.001; Wilcoxon rank-sum test) (Fig. 3a; Table S3); however, these differences were driven primarily by rare OTUs in soil samples, with Shannon and inverse Simpson indices were similar between sample locations (Fig. S3; Table S3). Beta diversity of the samples was analysed by weighted Unifrac and visualised on an nMDS ordination plot (Fig. 3b). PERMANOVA analysis confirmed communities significantly differed between sample locations (*p* = 0.001); communities in mound core and periphery samples were similar and compositionally distinct from soil communities, and methanotroph communities in soils beneath mounds were more similar to mounds than surrounding soils (Fig. 3b, c). In addition, methanotroph communities differed significantly between termite species (*p* = 0.022), while soil samples did not. Specifically, mound communities differed between Ms and Mn in both mound core (*p* = 0.005) and periphery (*p* = 0.032), and between Ms and Tp for mound core (*p* = 0.015).

The 25 OTUs detected were visualised on a phylogenetic tree (Fig. 2) against reference sequences from a curated *pmoA* gene database [34]. Across the mound and soil samples, over 80% of the sequences were affiliated with USCα, a recently cultivated lineage of alphaproteobacterial methanotrophs known to mediate atmospheric CH_4_ oxidation [70, 71] (Figs. 2 and 3c). The dominance of USCα was confirmed by less-resolved 16S rRNA gene and metagenome-based analyses, where they were estimated to comprise an average of 74% and 88% of the alphaproteobacterial methanotroph community, respectively (Fig. S4; Table S2); in both analyses, most sequences detected were closely related to those encoded by two USCα bacteria, *Candidatus* Methyloaffinis lahnbergensis [71] and *Methylocapsa gorgona* MG08 [70]. Based on *pmoA* amplicon sequencing and metagenome sequencing, the second most dominant taxonomic groups were affiliated with the alphaproteobacterial lineage *Methylocystis* in mound samples (<10% relative abundance) and the uncultivated tropical upland soil cluster (TUSC) [18] in soil samples (<10% relative abundance) (Fig. 2; Table S2). There was a large proportion of shared taxa across the samples, with the three most abundant OTUs (USCα-affiliated) present in all samples, regardless of type (mound vs soil), location and termite species (Fig. 3c). However, differential abundance analysis supported the differences between sample type and termite species observed by Unifrac analysis (Fig. 3b, c). USCα OTUs were more abundant in mound core, mound periphery and soils beneath, whereas TUSC OTUs were more abundant in surrounding soils. Significant differential abundance was also observed for certain OTUs between mounds of different termite species (Fig. 3b, c). Overall, these findings suggest that termite mounds contain methanotroph communities that are primarily derived from soil, but have a distinct composition overall.

### Methanotroph communities are kinetically adapted to elevated CH_4_ concentrations

**Fig. 4:**
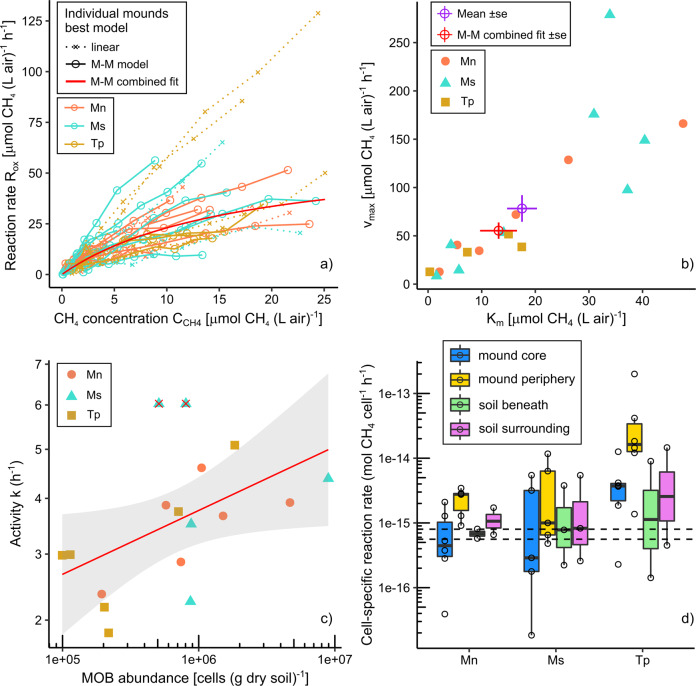
In situ kinetic parameters of CH_4_ oxidation in termite mounds. **a** Optimal Michaelis–Menten (M–M) curve estimated for a combined dataset of in situ concentrations and reaction rates from 18 out of 29 mounds showing M–M behaviour, excluding 11 mounds with an apparent linear increase of rates based on AIC. **b** Individual M–M parameters of the 18 mounds showing M–M behaviour, including their mean and standard error, and the optimal M–M parameters of the combined dataset. **c** Positive correlation of activity coefficients *k* with *pmoA* gene abundance in mound core samples of the 17 mounds selected for detailed analysis (*R*^2^ = 0.44, *p* = 0.0069). Two outliers have been removed, indicated with red X. **d** Cell-specific reaction rates of CH_4_ oxidation, estimated from in situ oxidation rates in termite mounds divided by methanotroph abundance at the different sampling locations. For each mound, the same oxidation rate was divided by four different abundances for comparison. The dashed horizontal lines indicate a range of literature values estimated in upland soils [29].

We determined the kinetics of CH_4_ oxidation in 29 mounds from all three species by performing in situ GPPTs. Methane oxidation rate was high across the investigated mounds (Fig. 4a). The relationship between CH_4_ concentration and reaction rate best fitted a Michaelis–Menten model for 18 mounds and a linear model for 11 mounds based on AIC values (Fig. 4a). For the former group of mounds, apparent Michaelis–Menten coefficients (*K*_m_, *V*_max_) were calculated. Estimated *K*_m_ values for the 18 mounds ranged from 0.32 to 47 µmol (l air)^−1^, and *V*_max_ from 8.4 to 280 µmol (l air)^−1^ h^−1^. These parameters did not significantly differ between mounds of different termite species. The overall mean values for *K*_m_ and *V*_max_ were 17.5 ± 3.5 µmol (l air)^−1^ and 78.3 ± 17 µmol (l air)^−1^ h^−1^, respectively (standard error of the mean); such values were close to the optimal parameters when fitting a Michaelis–Menten model to combined GPPT data (excluding mounds with linear behaviour): *K*_m_ = 13.2 ± 3.5 µmol (l air)^−1^ and *V*_max_ = 55.4 ± 8.5 µmol (l air)^−1^ h^−1^ (Fig. 4a, b). Thus, the methanotroph communities within termite mounds have an apparent medium (µM) affinity for CH_4_. The apparent *K*_m_ is approximately one to two orders of magnitude higher than high-affinity (nM) uptake observed in upland soils [72–74], but one to two orders of magnitude lower than the low-affinity (mM) uptake measured in landfill cover soils [75]. Similar Michaelis–Menten values were estimated from GPPTs in the vadose zone above a contaminated aquifer, which featured CH_4_ concentrations in a similar range to termite mounds (~1–40 μl l^−1^) [64].

It is noteworthy that 11 mounds showed an apparent linear increase of reaction rates with substrate concentrations (Fig. 4a). This could indicate that *V*_max_ has not been reached during GPPTs with a maximum injected CH_4_ concentration of ~900 µl l^−1^ (~40 µM); indeed, the injection concentration is in the range of our highest *K*_m_, thus the capacity of some mounds to oxidise CH_4_ can be substantially higher. A linear increase could also indicate a shift in kinetics during the course of the GPPT. It is in the nature of the GPPT that different areas of the mound are exposed to different concentration ranges, depending on their distance to the gas injection/extraction point [63]. Hence, gas extracted at different times may have had a “history” of exposure to methanotroph communities with different kinetics. It is even conceivable that the 1 h long exposure to high injection concentrations around the injection/extraction point triggered the upregulation of low-affinity methane monooxygenase isozymes, such as those reported in *Methylocystis* sp. SC2 [76].

For the 17 selected mounds investigated in more detail, we analysed how the kinetics and abundance of the methanotroph communities were correlated. While a range of positive correlations were observed (Table S3), the most robustly supported was the relationship between the first-order activity coefficient *k* and mound core methanotroph abundance (*R*^2^ = 0.40, *p* = 0.007) (Fig. 4c). This relationship also held true for total bacterial abundance (*R*^2^ = 0.36, *p* = 0.007). In contrast, correlations with methanotroph abundance in periphery and soils were not significant. These observations are in line with previous inferences that mound cores are primarily responsible for CH_4_ oxidation in *M. nervosus* and *M. sunteri* mounds [7]. On this basis, hypothetical cell-specific CH_4_ oxidation rates were calculated from in situ GPPT rates integrated across the whole mound, and divided by location-specific methanotroph abundances for comparing different mound and soil compartments. Mound core values ranged from 1.8 × 10^−17^ to 1.3 × 10^−14^ mol CH_4_ cell^−1^ h^−1^ (Fig. 4d), with the median of 1.3 × 10^−15^ mol CH_4_ cell^−1^ h^−1^ slightly higher than the observed range of 5–8 × 10^−^^16^ mol CH_4_ cell^−1^ h^−1^ in upland soils calculated by the same method [29]. This discrepancy was mainly driven by high cell-specific rates of *T. pastinator*, and the median dropped to 8.7 × 10^−^^16^ mol CH_4_ cell^−1^ h^−1^ when excluding this species. Also, cell-specific rates of periphery samples were unreasonably high for all species and particularly for *T. pastinator* (Fig. 4d), with some values close to landfill cover biofilters [77]. These high cell-specific rates in periphery (where there are significantly fewer methanotrophs) corroborate our findings of mound core methanotrophs being responsible of the bulk of the CH_4_ oxidation in mounds of *M. nervosus* and *M. sunteri* [7]. In addition, estimated rates for soils, particularly soil beneath mounds, were well aligned with upland soil literature values for all three species [29], and further emphasises that CH_4_ oxidation in *T. pastinator* mounds is driven by methanotrophs in the soil beneath.

### Termite mound USCα bacteria are predicted to be mixotrophic or facultative methanotrophs

**Fig. 5:**
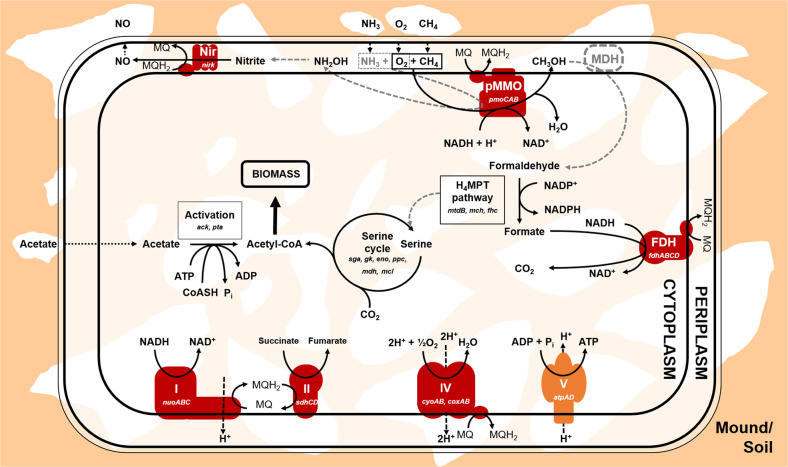
Metabolic reconstruction of the core pathways associated with energy conservation and carbon acquisition in the medium-quality metagenome-assembled genome (MAG-47) of a USCα methanotroph associated with *M. sunteri* mounds. H_4_MPT tetrahydromethanopterin pathway, pMMO particulate methane monooxygenase, MDH methanol dehydrogenase, FDH formate dehydrogenase, NiR copper-containing nitrite reductase, I NADH dehydrogenase (complex I), II succinate dehydrogenase (complex II), IV cytochrome *aa*_3_ oxidase and cytochrome *bo*_3_ oxidase (complex IV). Genes detected are shown in italics. Dashed black lines indicate diffusion. Dashed grey lines indicate reactions performed by three enzymes that were not detected, methanol dehydrogenase, hydroxylamine dehydrogenase and serine hydroxymethyltransferase. It is assumed that these enzymes are encoded by the bacterium, but were not detected given the moderate predicted completeness of the MAG (73%).

Finally, we performed metagenomic assembly and binning to gain a genome-resolved view of the taxonomic affiliation and metabolic capabilities of termite mound methanotrophs. We retrieved a medium-quality [78] metagenome-assembled genome (MAG-47; completeness 73%, contamination 1.7%) from pooled DNA extracted from *M. sunteri* mound cores. A genome tree confirmed this bacterium is a USCα alphaproteobacterium closely related to *Candidatus* Methyloaffinis lahnbergensis [71] and an Antarctic MAG [79] predicted to oxidise atmospheric CH_4_ (Fig. S5). This supports our conclusions from analysis of *pmoA* amplicons (Figs. 2 and 3), 16S rRNA gene amplicons (Fig. S4), and metagenomic short reads (Table S2) that USCα dominates the methanotrophic community of North Australian termite mounds. We inferred metabolic capabilities of the termite mound USCα based on the retrieved genome (Table S5). As depicted in Fig. 5, the bacterium encodes a particulate methane monooxygenase via a complete *pmoCAB* operon. The bacterium also has the capacity to assimilate carbon derived from CH_4_ through the H_4_MPT pathway and serine cycle, and conserve energy through aerobic respiration using two terminal oxidases. These core metabolic features are conserved with other recently sequenced USCα bacteria [70, 71].

It is likely that termite mounds are inhabited by mixotrophic or facultative methanotrophs. Consistently, the reconstructed USCα genome encoded enzymes to assimilate acetate into biomass (acetate kinase, phosphotransacetylase); previous studies have experimentally shown that USCα can assimilate acetate together with atmospheric CH_4_ [71, 80]. However, in contrast to a recent study that suggested *M. gorgona* can consume atmospheric H_2_ and CO [70], we did not detect hydrogenase or carbon monoxide dehydrogenase genes in the MAG; this may reflect niche differentiation between USCα methanotrophs or alternatively the incompleteness of the MAG. Nevertheless, homology-based searches of metagenome short reads of mound cores showed 2.8% of HhyL reads (group 1 h [NiFe]-hydrogenase) [81] and 0.03% of CoxL reads (type I carbon monoxide dehydrogenase) [82] were most closely related to those in alphaproteobacterial methanotroph genomes. This suggests that at least some methanotrophs in termite mounds consume atmospheric H_2_ and CO, in line with recent studies showing mixotrophic trace gas metabolism enables niche expansion of methanotrophs [70, 83, 84]. Finally, we detected a copper-containing nitrite reductase (NirK) in the MAG. It has recently been shown that this enzyme enables rapid detoxification of metabolites formed by the promiscuous oxidation of ammonia by particulate methane monooxygenase [85]. This process may be particularly important in termite mounds, given ammonia accumulates at high levels due to excretion by termites [23]; ammonia levels were particularly high in *M. sunteri* mounds from which the MAG was assembled (220 mg kg^−1^; Table S6).

## Conclusions and perspectives

Overall, our results imply that local environmental concentrations of CH_4_ shape the composition and kinetics of the methanotroph community in termite mounds. Elevated CH_4_ production from termites appears to have selected for a specialised methanotroph community within mounds with medium substrate affinity. The community analysis shows that the methanotroph communities within termite mounds are closely related to those of adjoining soils. However, termite activity has facilitated selection for USCα and *Methylocystis* OTUs, together with exclusion of TUSC OTUs, in mounds compared with adjoining soil. This selection is likely to be primarily driven by elevated CH_4_ availability, but other effects of mound formation and termite activity (e.g. on nutrient levels, moisture levels, temperature and vegetation cover) may also influence methanotroph community assembly. Concordant findings were made across three different species, with methanotroph communities showing high cell-specific reaction rates and medium affinities for CH_4_. They are thus ideally kinetically adapted to grow on termite-derived CH_4_ and in turn reduce atmospheric emissions of this greenhouse gas. These findings are also supported by the reconstruction of a metagenome-assembled genome of a USCα bacterium, confirming the capacity of mound methanotrophs to use CH_4_ as a carbon and energy source, together with acetate and potentially other substrates.

However, the apparent kinetic parameters of CH_4_ oxidation in termite mounds are atypical of the dominant groups of methanotrophs present. It is probable that USCα mediates most of the overall CH_4_ oxidation in the investigated mounds, given they were observed to be the most abundant and prevalent mound and soil methanotrophs across three separate analyses. This inference is also reflected by consistent cell-specific rates of CH_4_ oxidation in mound core and soil. However, USCα are typically high-affinity methanotrophs associated with the oxidation of CH_4_ at atmospheric concentrations in upland soils [70, 71]. There are several possible explanations for this apparent discrepancy. Selective pressures may have caused mound USCα to modulate their apparent CH_4_ affinity, for example through amino acid substitutions or gene expression changes of their particulate methane monooxygenases; the first explanation is unlikely given soil and mound USCα encode common PmoA subunits, though substitutions in PmoB or PmoC cannot be ruled out. An alternative explanation is community heterogeneity. Any apparent Michaelis–Menten parameter estimates from environmental samples are “bulk” values integrating across the whole microbial community and thus a vast array of potentially different enzymes. Indeed, the observed in situ kinetics are also compatible with the coexistence of slow-acting, high-affinity USCα methanotrophs alongside faster-acting, low-affinity other groups. *Methylocystis*-like OTUs, as the second most abundant group, are likely to be particularly competitive at higher CH_4_ concentrations during phases of high termite activity. Members of this group encode kinetically distinct methane monooxygenase isozymes [76] and have been identified in other soils with elevated CH_4_ concentrations [22]. They would therefore have a competitive advantage in termite mounds where CH_4_ availability is elevated and exhibits considerable temporal and spatial variation. Lastly, while CH_4_ concentrations in mounds are elevated compared with the atmosphere (2–100 ppmv) [7, 86], they are well within the accessible range for high-affinity methanotrophs such as USCα.

Despite elevated substrate availability, methanotrophs remain minor members of microbial communities in termite mounds at our sampling sites. We observed that methanotrophs comprised <0.5% of the bacterial community across three separate assays, although the exact proportion varied depending on the method used. While methanotrophs are moderately enriched in mound core compared to adjoining soil overall, they are actually diminished relative to soil in both the mound core of *T. pastinator* and the periphery of all three species. The observed strong correlation of methanotroph abundance with the porosity of the mound material for all species strengthens the case for habitat porosity as a crucial factor in regulating the methanotrophic community, likely by regulating local CH_4_ availability through diffusion. Another factor driving low methanotroph abundance could be the accumulation of ammonia, which is produced in high levels by termites [23]; it is known that ammonia competitively inhibits methane monooxygenase activity [87, 88] and ammonia levels are major environmental factors regulating methanotroph community in soils [89, 90]. However, the high ammonia concentrations in mounds of *M. sunteri* (Table S6) did not correlate with interspecies differences in methanotroph abundance, yet aligned well with differences in community composition (Fig. 3). High ammonia levels may potentially drive adaptation of the methanotroph community, rather than diminishing abundance, as supported by the potential mechanism to detoxify ammonia-derived intermediates in the USCα MAG found in *M. sunteri* mounds. Putative ammonia-oxidising bacteria and archaea were also detected in the 16S rRNA gene datasets, but like methanotrophs, were in relatively low abundance (<0.5%) despite substrate availability and hence are unlikely to mediate significant rates of nonspecific CH_4_ oxidation. Other possible factors limiting methanotrophs in termite mounds, such as the micronutrients (e.g. copper) required for methane monooxygenase activity, remain to be investigated.

It should be noted that it is unclear to what extent these observations are extendable to mounds from other regions or species. Broadly, our findings agree with a previous study that inferred mounds of the African fungus-growing termite *Macrotermes falciger* have a distinct methanotroph community from surrounding soil, likely in part due to selective pressures exerted by termite CH_4_ emissions [14]. However, given methanotroph communities are derived from surrounding soil, the specific communities mitigating CH_4_ uptake are likely to greatly vary between different soil types. Indeed, we observed a striking difference in community composition of the mounds from the three Australian termite species compared with those of the one African termite species [14]. The African mounds were dominated by the JR3 cluster, a gammaproteobacterial methanotroph lineage closely related to USCγ, which was detected in very low abundance in the Australian mounds (Fig. 2; Table S2). These differences likely reflect the distinct habitat specificity of USCγ and USCα methanotrophs. USCα often occurs in acidic to neutral upland soils ([67], reviewed in [18]), which match the pH values between 5 and 6 measured in the mounds of this study (Table S3). In contrast, USCγ and associated lineages are commonly found in upland soils of neutral to basic pH [91, 92], which corresponds well to pH values of 7–8 in *Macrotermes falciger* mounds [14]. Global sampling would be required to more broadly determine how soil and termite characteristics influence the methanotroph community and CH_4_ oxidation kinetics of termite mounds. Our findings are nevertheless likely to extend to many Australian mounds, given the sampling site is broadly representative of much of tropical northern Australia [7, 93] and the three termite species selected represent the three major feeding groups on the continent. Overall, we conclude that methanotrophs remain minor members of mounds at our sampling sites and hence are only able to mitigate a proportion of the large amounts of CH_4_ produced by mound-dwelling termites.

## Supplementary information

Supplementary information

Table S2

Table S3

Table S4

Table S5

## Data Availability

The sequence dataset generated and/or analysed during the current study are publicly available under NCBI BioProject accession number PRJNA641804. All metadata is provided in the supplemental material.
