# Supplementary material for: Termite mounds contain soil-derived methanotroph communities kinetically adapted to elevated methane concentrations
Source: ISME J. 2020 Jul 24;14(11):2715–31. doi: 10.1038/s41396-020-0722-3 (PMC7784690; doi:10.1038/s41396-020-0722-3)

Supplemental material

Termite mounds contain soil-derived methanotroph communities kinetically adapted to elevated methane concentrations

Eleonora Chiri[1,2,3 #], Chris Greening[1,3 *], Rachael Lappan[1,3], David W. Waite[4], Thanavit Jirapanjawat[1,3], Xiyang Dong[5], Stefan K. Arndt[2 *], Philipp A. Nauer[2,6 #]

[1] Department of Microbiology, Biomedicine Discovery Institute, Monash University, Clayton, VIC 3800, Australia
[2] School of Ecosystem and Forest Sciences, University of Melbourne, Richmond, VIC 3121, Australia
[3] School of Biological Sciences, Monash University, Clayton, VIC 3800, Australia
[4] School of Biological Sciences, University of Auckland, Auckland, New Zealand
[5] School of Marine Sciences, Sun Yat-Sen University, Zhuhai 519082, China
[6] School of Chemistry, Monash University, Clayton VIC 3800, Australia
[#] These authors contributed equally to this work.

* Correspondence can be addressed to:

Assoc Prof Chris Greening (chris.greening@monash.edu), Department of Microbiology, Monash University, Clayton, VIC 3800, Australia

Prof Stefan Arndt (sarndt@unimelb.edu.au), School of Ecosystem and Forest Sciences, University of Melbourne, Richmond, VIC 3121, Australia

Table S1. List of the 16S rRNA gene sequences included in the reference database.

**Microorganism**	**Accession number**
Beijerinckia indica ATCC 9039 (CP001016)	NR_074269.1 (RefSeq)
Beijerinckia mobilis (AB119200)	AB119200.1 (ENA)
Methyloferula stellata AR4 (FR686343)	FR686343.1 (GenBank)
MFS USCα (*Candidatus* Methyloaffinis lahnbergensis)[a]	MG203879.1 (GenBank)
Methylocapsa palsarum NE2 (KP715289)	KP715289.1 (GenBank)
Methylocapsa aurea KYGT (JQKO01000009)	FN433469.1 (GenBank)
Methylocapsa acidiphila B2 (AJ278726)	AJ278726.1 (GenBank)
Methylocella palustris Y5 (AJ563925)	AJ563925.1 (GenBank)
Methylocella silvestris BL2 (AJ491847)	AJ491847.1 (GenBank)
Methylosinus trichosporium OB3b (ADVE02000001)	NR_044947.1 (RefSeq)
Methylocystis parvus (AF150805)[b]	AF150805.1 (GenBank)
Methylocystis bryophila H2sT (FN422003)	FN422003.1 (GenBank)
Chelatococcus asaccharovorans CP141b (AJ871433)	AJ871433.1 (GenBank)
Bradyrhizobium japonicum NA6545 (AB070562)	AB070562.1 (GenBank)
Hyphomicrobium denitrificans LWQ79 (KC854850)	KC854850.1 (GenBank)
Xanthobacter autotrophicus NBRC 14758 (AB680655)	AB680655.1 (GenBank)
Uncultured freshwater wetland clone (JX505201)	JX505201.1 (GenBank)
Uncultured temperate grassland clone (JN023555)[a]	JN023555.1 (GenBank)
Uncultured lava tube wall biofilm clone (HM445356)	HM445356.1 (GenBank)
Uncultured forest soil clone (AY913393)	AY913393.1 (GenBank)
Uncultured cave wall biofilm clone (DQ823224)[a]	DQ823224.1 (GenBank)
Methylocapsa gorgona strain M608	CP024846.1:2611277-2612782 (GenBank)
Beijerinckia doebereinerae LMG 2819 (NR_116304)	NR_116304.1 (RefSeq)
Beijerinckia mobilis DSM 2326 (NR_042180)	NR_042180.1 (RefSeq)
Methylocella tundrae T4 (NR_025596)	NR_025596.1 (RefSeq)
Methylocystis echinoides IMET 10491 (NR_025544)[b]	NR_025544.1 (RefSeq)
Methylococcus capsulatus Texas (NR_029241)	NR_029241.1 (RefSeq)
Methylmicrobium album ACM 3314 (X72777)	X72777.1 (GenBank)
Methylobacter luteus NCIMB 11914 (AF304195)	AF304195.1 (GenBank)
Methylomonas methanica S1 (AF304196)	AF304196.1 (GenBank)
NC10 Candidatus Methylomirabilis oxyfera	FJ621559.1 (GenBank)
Verrucomicrobia bacterium (Methylacidiphilum infernorum)	AM900833.1 (GenBank)
Methylacidiphilum fumariolicum strain SolV	EF591088.1 (GenBank)

[a] These microorganisms are identical in the V4 region of the 16S rRNA gene
[b] These microorganisms are identical in the V4 region of the 16S rRNA gene

Table S2 (xlsx). Summary of PmoA and MmoX reads detected by shotgun metagenome sequencing.

Table S3 (xlsx). Summary table of physical data, kinetic parameters, abundance, and diversity data for all investigated mounds, as well as statistical tests performed.

Table S4 (xlsx). Sequences and counts of all denoised amplicon sequence variants (ASVs) resolved by 16S rRNA gene sequencing.

Table S5 (xlsx). Protein sequences and annotations predicted from the termite mound methanotroph metagenome-assembled genome (MAG-47).

Table S6. Soil physicochemical data for termite mound core, termite mound periphery, and soil samples. A composite sample was analysed for each species (*Tumulitermes pastinator*, Tp; *Microcerotermes nervosus*, Mn; *Macrognathotermes sunteri*, Ms) and location (mound core, c; mound periphery, p; soil).

	pH (H_2O)	TOC	TC	TN	NH_4^+-N	NO_3^--N	PO_4^{2-}	K^+
		% (kg kg^{-1})			mg kg^{-1}			
Tp-p	5.4	1.86	2.05	0.09	16	6	6	99
Tp-c	5.4	2.11	2.36	0.10	15	6	5	106
Mn-p	5.5	5.04	7.15	0.25	32	9	9	187
Mn-c	5.8	4.83	6.68	0.24	39	3	8	206
Ms-p	5.2	5.05	9.96	0.42	280	80	40	140
Ms-c	5.2	4.41	10.7	0.44	220	36	29	128
Soil	5.7	2.38	3.25	0.20	22	7	10	50

Figure S1. Abundance of total bacteria and methane-oxidising bacteria (MOB, methanotrophs) in the mounds and adjoining soils. a) Abundance of the total microbial community, based on copy number of the universal 16S rRNA gene as determined by qPCR, and assuming an average of 4.2 copies per cell. b) Abundance of the methanotroph community, based on copy number of the *pmoA* gene encoding the particulate methane monooxygenase 27 kDa subunit gene as determined by qPCR, and assuming 2 copies per cell. c) Abundance of the methanotroph community, based on the fraction of methanotrophs estimated from 16S rRNA gene sequencing. The box plots show minimum, lower quartile, median and upper quartile, with all individual values shown. The dashed lines in d) represent mean fractions of methanotrophs vs total bacterial abundance for *pmoA*-based methanotroph abundance estimates (blue), 16S rRNA-based estimates (brown) and metagenome-based estimates (green).

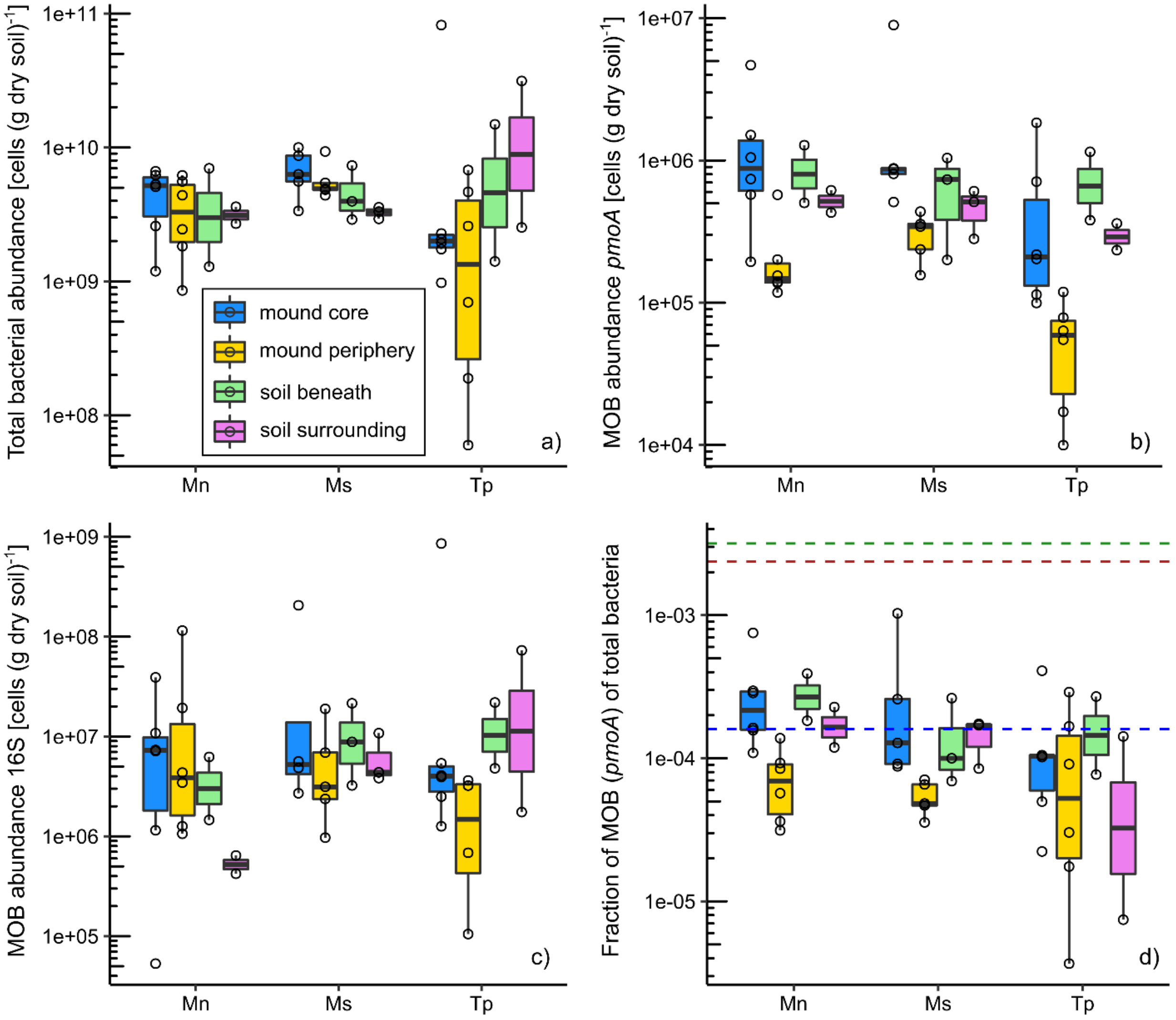

Total bacterial abundance [cells (g dry soil)^-1]
1e+11
1e+10
1e+09
1e+08
mound core
mound periphery
soil beneath
soil surrounding
a)
Mn
Ms
Tp
MOB abundance pmoA [cells (g dry soil)^-1]
1e+07
1e+06
1e+05
1e+04
b)
MOB abundance 16S [cells (g dry soil)^-1]
1e+09
1e+08
1e+07
1e+06
1e+05
c)
Fraction of MOB (pmoA) of total bacteria
1e-03
1e-04
1e-05
d)

Figure S2. Summary of the 16S rRNA gene-based analysis of microbial communities from four locations (mound core and periphery; soil beneath and surrounding the mound) and three termite species (*Microcerotermes nervosus*, *Macrognathotermes sunteri*, *Tumulitermes pastinator*). a) Observed richness (left), Shannon diversity (middle), and Inverse Simpson diversity (right) of the community. b) Non-metric multidimensional scaling (NMDS) ordination of the microbial community structure (beta diversity) measured by Bray-Curtis distance matrix of 16S rRNA gene amplicon sequences. c) Average relative abundance (percent) of 16S rRNA gene amplicon sequences resolved at the taxonomic level of class. The depicted values represent the average relative abundance of the methanotrophic ASVs, all of which affiliate with the class Alphaproteobacteria based on phylogenetic analysis.

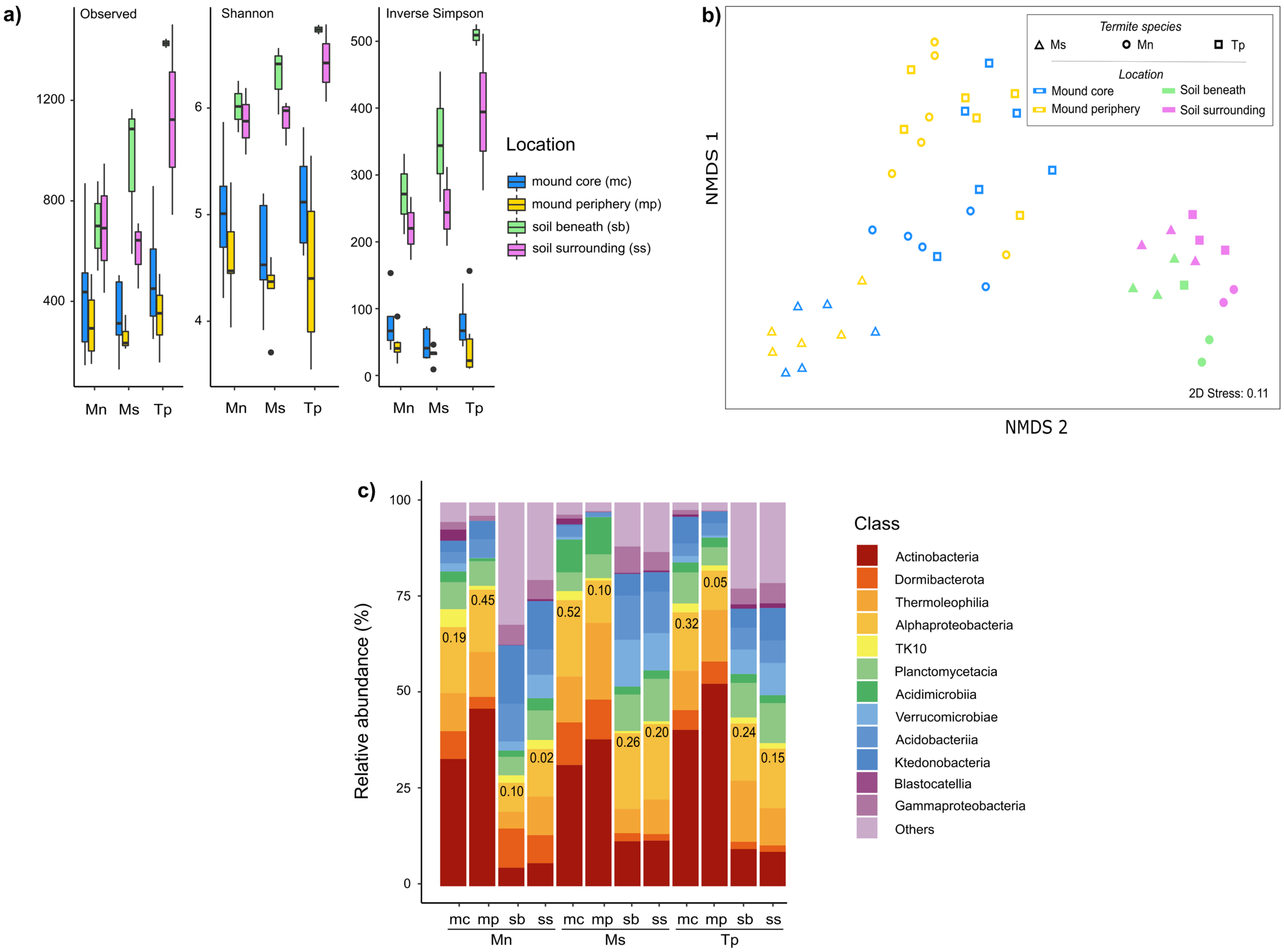
a)
Observed
Shannon
Inverse Simpson
Mn Ms Tp
Location
mound core (mc)
mound periphery (mp)
soil beneath (sb)
soil surrounding (ss)
b)
Termite species
Ms
Mn
Tp
Location
Mound core
Mound periphery
Soil beneath
Soil surrounding
NMDS 1
NMDS 2
2D Stress: 0.11
c)
Relative abundance (%)
0.19 0.45 0.10 0.02 0.52 0.10 0.26 0.20 0.32 0.05 0.24 0.15
mc mp sb ss mc mp sb ss mc mp sb ss
Mn
Ms
Tp
Class
Actinobacteria
Dormibacterota
Thermoleophilia
Alphaproteobacteria
TK10
Planctomycetacia
Acidimicrobiia
Verrucomicrobiae
Acidobacteriia
Ktedonobacteria
Blastocatellia
Gammaproteobacteria
Others

Figure S3. Estimated diversity of methanotrophs within different termite mound and soil samples, based on amplicon sequencing of the *pmoA* gene. a) Shannon diversity, and b) Inverse Simpson diversity, grouped according to species and sampling location. Mound samples (core and periphery) and corresponding soil samples (surrounding and beneath mound) were tested for differences among all species. No significant differences were observed between sample groups (all $p > 0.05$, Kruskal-Wallis test).

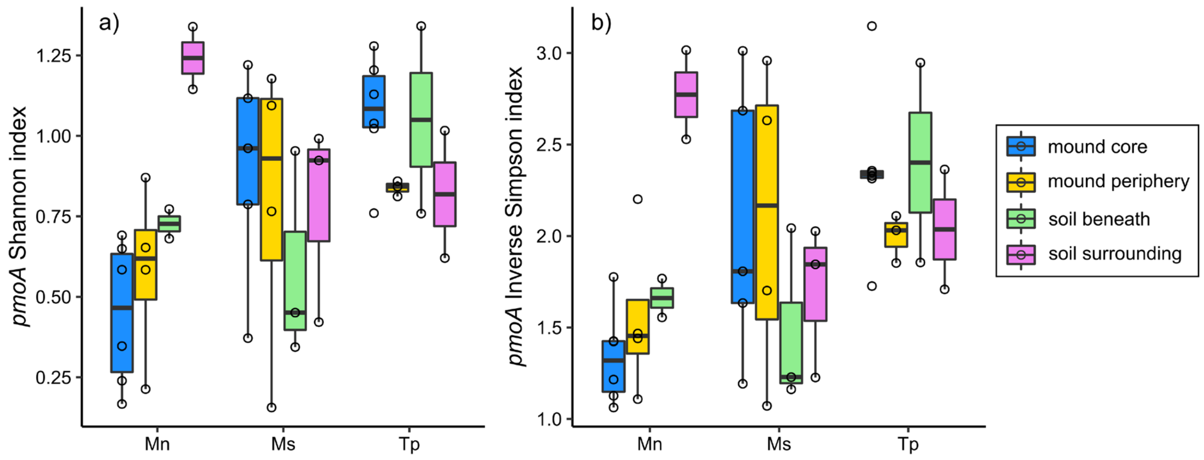

Figure S4. Maximum-likelihood tree showing the phylogenetic affiliation of the nucleotide sequence of 27 amplicon sequence variants (ASVs) of the V4 hypervariable region of the 16S rRNA gene predicted to be methanotrophs. The 27 ASVs assigned to methanotrophs are depicted in bold and numbered according to decreasing relative abundance among all samples. Non-methanotrophic reference sequences are depicted in red. Genebank accession numbers for the sequences at individual node tips are given in parentheses. The tree was constructed using the GTR-based model and was bootstrapped with 100 replicates. Node numbers indicate bootstrap branch support ≥ 30. The scale bar displays 0.08 changes per nucleotide position.

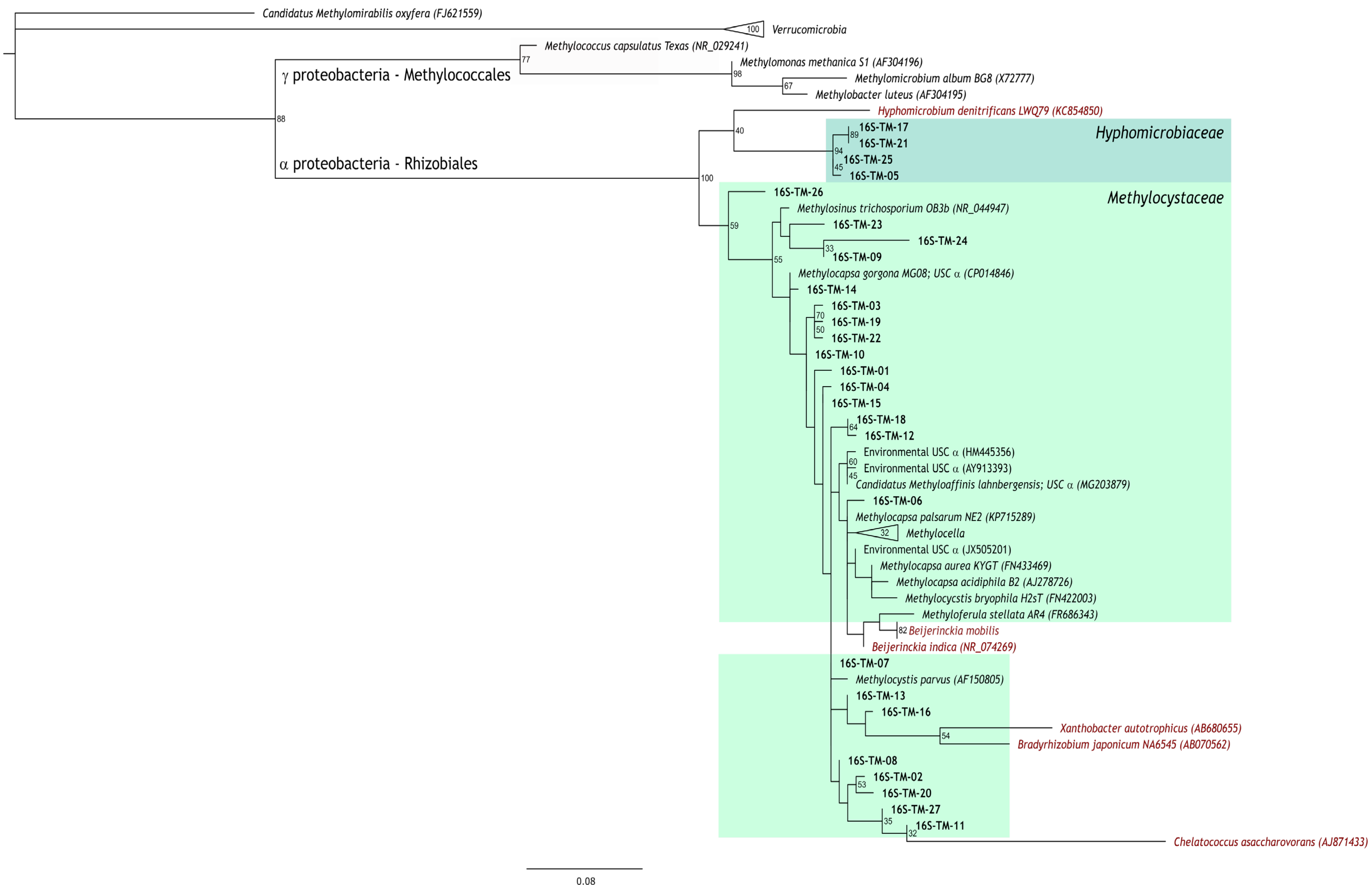

Candidatus Methylomirabilis oxyfera (FJ621559)
100 Verrucomicrobia
Methylococcus capsulatus Texas (NR_029241)
77
γ proteobacteria - Methylococcales
Methylomonas methanica S1 (AF304196)
98
Methylomicrobium album BG8 (X72777)
67
Methylobacter luteus (AF304195)
88
Hyphomicrobium denitrificans LWQ79 (KC854850)
40
Hyphomicrobiaceae
89 16S-TM-17
16S-TM-21
94
16S-TM-25
45
16S-TM-05
α proteobacteria - Rhizobiales
100
16S-TM-26
Methylocystaceae
Methylosinus trichosporium OB3b (NR_044947)
59
16S-TM-23
16S-TM-24
33
16S-TM-09
55
Methylocapsa gorgona MG08; USC α (CP014846)
16S-TM-14
16S-TM-03
70
16S-TM-19
50
16S-TM-22
16S-TM-10
16S-TM-01
16S-TM-04
16S-TM-15
16S-TM-18
64
16S-TM-12
Environmental USC α (HM445356)
60
Environmental USC α (AY913393)
45
Candidatus Methyloaffinis lahnbergensis; USC α (MG203879)
16S-TM-06
Methylocapsa palsarum NE2 (KP715289)
32 Methylocella
Environmental USC α (JX505201)
Methylocapsa aurea KYGT (FN433469)
Methylocapsa acidiphila B2 (AJ278726)
Methylocycstis bryophila H2sT (FN422003)
Methyloferula stellata AR4 (FR686343)
82 Beijerinckia mobilis
Beijerinckia indica (NR_074269)
16S-TM-07
Methylocystis parvus (AF150805)
16S-TM-13
16S-TM-16
Xanthobacter autotrophicus (AB680655)
54
Bradyrhizobium japonicum NA6545 (AB070562)
16S-TM-08
53
16S-TM-02
16S-TM-20
16S-TM-27
35
16S-TM-11
32
Chelatococcus asaccharovorans (AJ871433)
0.08

Figure S5. Genome tree comparing the phylogenetic placement of MAG-47 with reference genomes from the order *Rhizobiales*. Tree inference was performed using the LG+G model of protein substitution with 100 bootstrap supports to assess node stability. Support is represented by solid (>90%) and hollow (>70%) junctions. MAG-47 is bolded and the scale bar shows 0.1 substitutions per base.

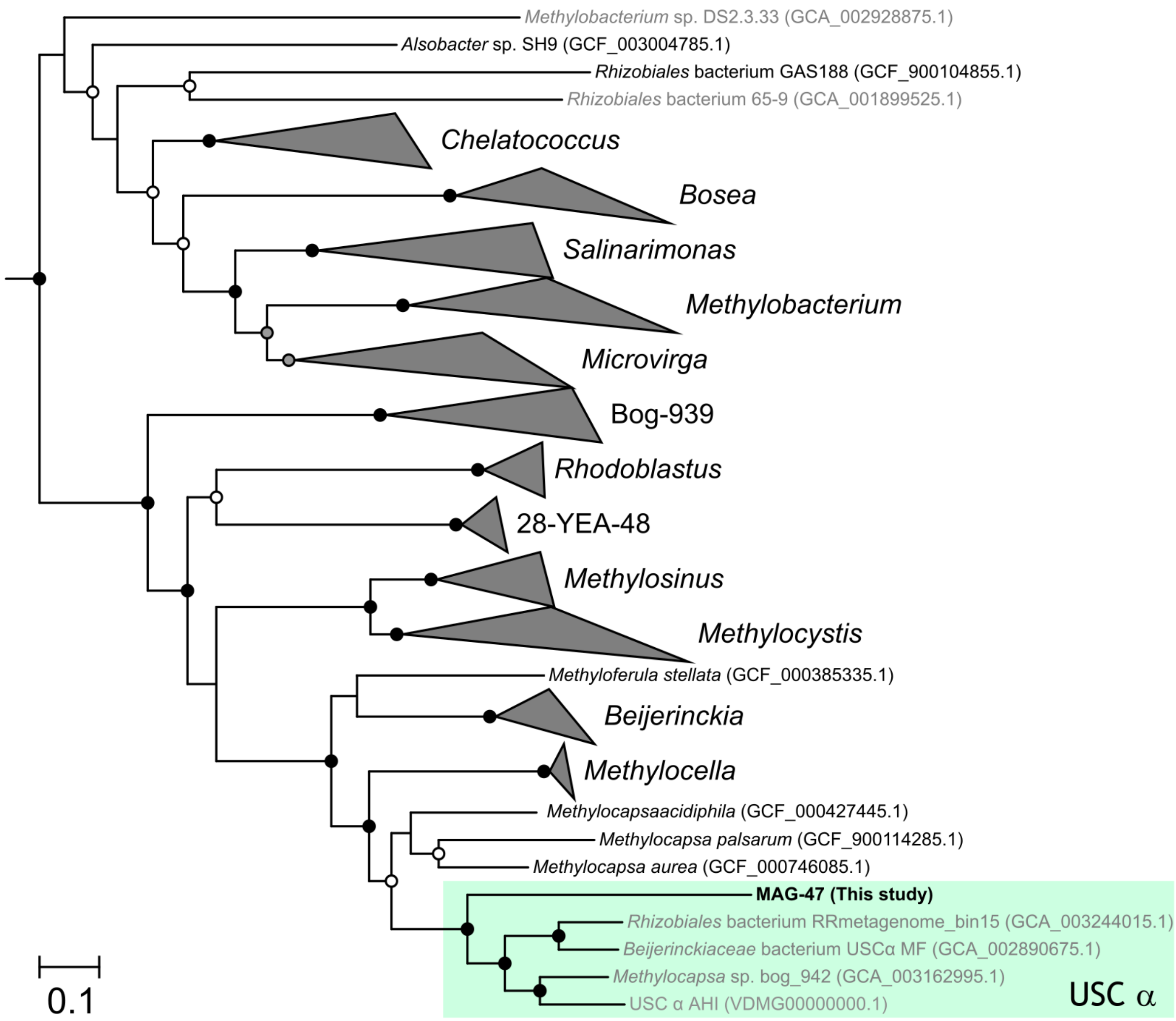

Supplement: Supplementary file 1 — Supplementary information [file 41396_2020_722_MOESM1_ESM.pdf]
